# Indicators and medical tests to identify lower limb swelling causes after total knee arthroplasty: a Delphi study with multidisciplinary experts

**DOI:** 10.1186/s13018-023-03980-6

**Published:** 2023-08-05

**Authors:** Lin Yang, Bei-Ying Wu, Cai-feng Wang, Hui-Wu Li, Wei-Wei Bian, Hong Ruan

**Affiliations:** 1 Department of Nursing, Shanghai Ninth People’s Hospital, Shanghai JiaoTong University School of Medicine, Shanghai, China; 2 School of Nursing, Shanghai JiaoTong University, Shanghai, China; 3 Department of Orthopedic, Shanghai Ninth People’s Hospital, Shanghai JiaoTong University School of Medicine, Shanghai, China

**Keywords:** Total knee arthroplasty, Swelling, Classification, Laboratory tests, Ultrasonography, Delphi method

## Abstract

**Background:**

Lower limb swelling after total knee arthroplasty (TKA) hinders surgical effectiveness. The poor results of studies on swelling interventions are due to the lack of a classification of swelling causes through appropriate medical tests. A gold standard is missing. This study aimed to clarify the causes of TKA postoperative swelling and how to identify them through indicators and medical tests by consulting a wide range of experts from multiple disciplines.

**Method:**

The Delphi method was used. A first draft of the index was prepared based on a systematic search of the literature. A total of 11 experts from several disciplines were invited to evaluate the rationality of the indicators and suggest modifications. After two rounds of consultation, the experts reached a consensus, and the consultation was stopped.

**Results:**

The response rate of the 11 experts was 100%, and the authoritative Cr was 0.896. Kendall's W values for opinion coordination of the two rounds of consultation were 0.262 and 0.226, respectively (P < 0.001). Among the final indicators, there were 4 primary indicators for swelling cause classification (inflammatory response, poor venous return, joint hematoma, muscle damage, and healing), 19 secondary and 19 tertiary indicators.

**Conclusion:**

The indications obtained by systematic literature review and multidisciplinary expert consultation are reliable and scientific. Multiple causes of lower extremity swelling after TKA were identified. Blood test indicators can reflect an inflammatory response, suggest poor venous return, and reflect muscle damage and healing progress. Ultrasound scans are needed to identify underlying thrombotic or valvular problems, joint hematomas, and muscle damage. These tests help clinicians and researchers determine the cause of swelling after TKA and take appropriate management.

## Background

TKA is an effective treatment for end-stage knee osteoarthritis. The number of procedures continues to grow rapidly worldwide, and only in the United States, TKA is expected to reach 3.41 million interventions/year by 2040 [1]. However, swelling of the lower limb after TKA affects up to 90.7% of patients at 2–3 weeks after discharge, lasting even up to 90 days postoperatively or longer [2]. Swelling can seriously affect the rehabilitation process and outcomes and can pose the following problems: (i) impaired muscle activation and strength: swelling due to joint-derived muscle inhibition leads to impaired quadriceps activation and decreased extensor strength, resulting in delayed rehabilitation [3, 4]; (ii) restricted joint movement and hindered exercises: swelling restricts joint flexion, hindering rehabilitation exercises and affecting early postoperative joint mobility [3]. It also has long-term effects on patients' gait speed [5]. (iii) Skin-related complications: severe swelling can cause blisters, skin breakdown, and increase the risk of joint infection; (iv) patient experience and satisfaction: swelling after TKA raises concerns for patients, resulting in a negative experience and low satisfaction levels. Therefore, swelling after TKA needs to be prevented with early intervention to improve the postoperative outcome.

Most previous studies on swelling after TKA have evaluated changes in knee circumference to reflect the pattern of swelling occurrence and intervention effects but neglected to analyze the causes of swelling specifically. Researchers have conducted intervention studies targeting various causes of swelling, such as reducing the inflammatory response resulting from intraoperative injury [6], constraining blood vessels to minimize knee bleeding [7], and enhancing lower limb venous return [8, 9]. However, some of these interventions did not achieve statistically significant results [7, 8, 10]. The different mechanisms of swelling interventions suggest the existence of multiple causes, which need to be accurately identified to maximize prevention and early intervention.

Literature review and comprehensive analysis of the anatomy of the knee, TKA procedure, and perioperative treatment revealed multiple causes of postoperative swelling, including inflammation, poor venous return, joint cavity hematoma, intracapsular synovial fluid, muscle injury and healing, ligament injury, and healing. However, it remained unclear which tests could help distinguish among them. This study aimed to clarify the causes of TKA postoperative swelling and how to identify them through indicators and medical tests by consulting a wide range of experts from multiple disciplines.

## Methods

The Delphi method was adopted in the present research [11]. To ensure methodological rigor and transparency in conducting the Delphi study, we adhered to the recommendations provided by the CREDES (Guidance on Conducting and Reporting Delphi Studies) [12].

### Research team

The research team included an orthopedic nurse leader, an orthopedic director, a nursing management specialist, and two orthopedic nurses. They were responsible for literature search, questionnaire preparation and distribution, compilation of statistics, analysis of expert opinions, and revision of entries.

### Development of the first draft of the indicators

Several databases, including PubMed, Web of Science, CINAHL, China National Knowledge Internet (CNKI), Wanfang Data and China Science and Technology Journal Database (Sinomed) were systematically searched to review the literature on postoperative swelling after TKA. The search terms included: ("knee replacement" OR "knee arthroplasty") AND ("swell*" OR "edema"); all publication years were included up to November 20, 2022. Intervention studies reporting causes and factors of TKA postoperative swelling after TKA were examined, and the first-level indicators of the draft was obtained. Since indicators and medical tests for different causes of swelling after TKA differ, the first-level indicators were classified as different causes of swelling. A further literature search was conducted, using search terms related to different causes of swelling, including: "inflammation" OR "thrombosis" OR "embolism" OR "muscle injury" OR "muscle healing" OR "hematoma" OR "ligament injury." Literature on medical tests for each type of swelling was reviewed, and the secondary and tertiary indicators were formed in the indicator draft. Secondary indicators were medical tests for each cause, and if these indicators required more detail, they were placed among tertiary indicators.

**Fig. 1:**
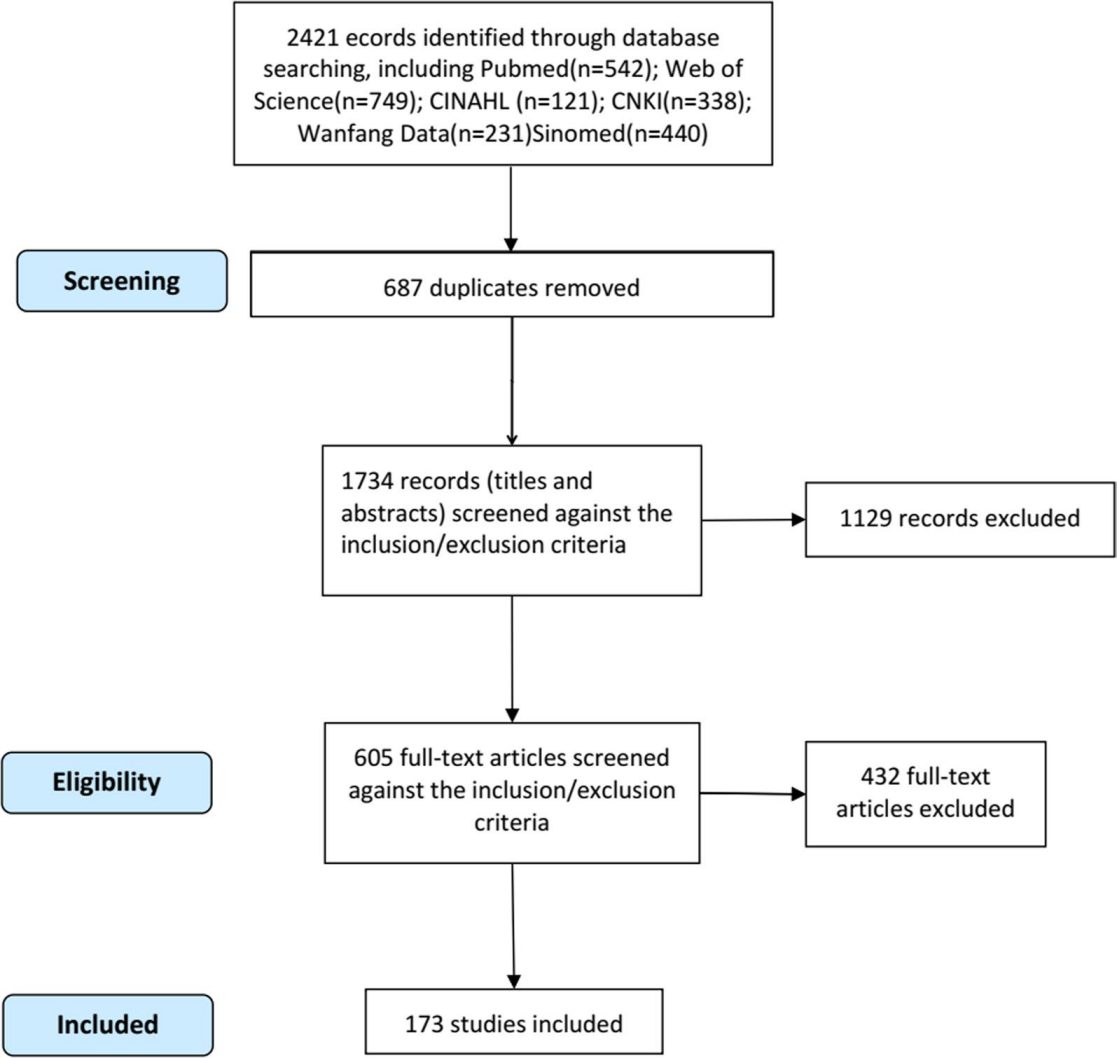
Process and results of literature search and screening (PRISMA Flow Diagram)

Inclusion criteria for the literature were as follows: (i) studies on lower limb swelling after TKA, (ii) studies that addressed the cause or mechanism of swelling or described the medical test for a specific mechanism, and (iii) studies published in Chinese or English language. Exclusion criteria included duplication studies and the ones with unavailable full text. The process and results of the literature search and screening are detailed in Fig. 1. After the research team analyzed the literature results, the first draft of the indicators was determined.

### Development of expert consultation questionnaire

The questionnaire distributed to experts for consultation contained three sections (Table 1).

**Table 1:** Content of expert consultation questionnaire

Sections	Content
Section 1: Survey Introduction	(1) Study overview: background, purpose, and significance of the study (2) Indicator development: the process of constructing the first draft of the indicators (3) Expert consultation: including the purpose of expert consultation, questionnaire components, expected return time, and contact information of the researcher
Section 2: Expert Assessment and Recommendations for Indicators	(1) First draft of the indicators (2) Reasonability of the indicators was evaluated by experts using a 5-point Likert scale: Very Reasonable (5 points), Reasonable (4 points), Fair (3 points), Not Very Reasonable (2 points), Unreasonable (1 point) (3) Modification Comments: comments from experts on any indicators are invited, including adding, removing, or modifying indicators
Section 3: Experts' Information and Self-evaluation	(1) Basic information of experts: including age, gender, department, years of work, highest education, technical title, whether postgraduate supervisors, and main research fields (2) Consensus (Cs): Self-evaluation of the expert's familiarity with the consultation content. Scores were assigned as follows: very familiar (0.9), more familiar (0.7), generally familiar (0.5), less familiar (0.3), and unfamiliar (0.1) (3) Criterion of Appropriateness (Ca): Self-evaluation by experts of the basis for their judgment in expressing their opinions. Scores were assigned as follows: practical experience (0.5, 0.4, 0.3), theoretical analysis (0.3, 0.2, 0.1), references (0.1, 0.1, 0.1), and intuition (0.1, 0.1, 0.1)

### Selection of experts for consultation

Experts from multiple fields were involved in consultation, including those in orthopedics, rehabilitation medicine, laboratory, ultrasound, radiology, and vascular surgery. Specialists in orthopedics and vascular surgery were incorporated in both the physicians and senior nurses. The experts had to meet the following criteria: (i) extensive practical or research experience in the fields related to TKA postoperative swelling, with a minimum of 15 years of working experience; (ii) bachelor's degree and above; (iii) intermediate technical title and above; (iv) motivation for this study; and (v) following the principle of informed consent.

### Implementation of expert consultation

The principal investigators (YL and RH) contacted experts by email or WeChat, distributing questionnaires and reminding them to return them within 2 weeks. The researcher contacted experts who did not respond after 2 weeks to learn about the progress and the reasons for the lack of response. After the questionnaires from the first round were returned, the researcher (YL) analyzed the experts' opinions and organized the research team discussion. Entries were removed according to the criteria of mean score of the importance of entries < 3.5 and coefficient of variation (CV) > 0.25, and entries were added, removed, and modified according to the experts' feedback. Based on the results of the first round of consultation, a second round of expert consultation questionnaires was designed. The results of the first round were provided in the second round of questionnaires so that the experts could understand how these modifications were created. The same steps were followed to distribute and collect the questionnaires to the experts and analyze the results. The consultation was concluded when the experts reached a consensus. Therefore, two rounds of expert consultation were conducted. The interval between the two rounds of expert consultation was one month. During this time, the research team compiled the data and formed the questionnaire for the second round of consultation.

### Statistical analysis

SPSS 22.0 software was used to analyze the data. The basic expert profile was expressed as mean, standard deviation, or frequency and percentage. The expert positivity coefficient was expressed using the questionnaire return rate. The degree of expert authority was expressed using the expert authority coefficient (Cr) as the arithmetic mean of Cs and Ca. The degree of expert opinion concentration was expressed by the mean importance score and CV. The degree of expert opinion coordination was expressed by Kendall's W coefficient.

## Results

A total of 11 experts were finally consulted by letter. The age was 38–59 years old, with an average of (48.64 ± 6.15) years. The working years were 15–38 years, with an average of (25.00 ± 7.92) years. The basic information of the experts is detailed in Table 2.

**Table 2:** Basic information of experts (n = 11)

Items	Groups	n	Percentage(%)
Gender	Men	7	63.6
	Women	4	36.4
Ages	≤ 40 years old	2	18.2
	41–50 years old	5	45.5
	51–60 years old	4	36.4
Education	Bachelor's degree	3	27.3
	PhD	8	72.7
Technical title	Intermediate	2	18.2
	Associate senior	2	18.2
	Full Senior	7	63.6
Years of work	15–20 years	4	36.4
	21–30 years	2	18.2
	31 years and above	5	45.5
Research/work area	Orthopedics	3	27.3
	Technical departments	3	27.3
	Rehabilitation	2	18.2
	Nursing	2	18.2
	Vascular surgery	1	9.1
Graduate student mentor	Doctoral Supervisor	3	27.3
	Master's degree advisor	8	72.7

### Degree of expert motivation

The positive degree of experts was expressed by the questionnaire return rate. The return rate was 100%. In the first round, 5 experts (45.5%) made 23 suggestions for revision. In the second round, 8 experts (72.3%) made 22 suggestions. This reflected the experts' interest in the study and the relatively high level of motivation.

### Degree of expert authority

The Cs of expert consultation was 0.81, Ca was 0.98, and Cr was 0.90, all of which were > 0.7, suggesting that the obtained results were reliable.

### Degree of expert opinion coordination

In this study, the importance scores of the two rounds of expert consultation were 3.09–4.82 and 3.18–4.82, CVs were 0.08–0.41 and 0.08–0.38, respectively. Kendall's W for the two rounds of expert consultation was 0.26 and 0.23 (P < 0.001 for both), indicating consistent expert opinions.

### Modification of the indicators

During the first round of expert consultation, 5 experts (45.5%) proposed 23 revisions. The indicators were revised based on the indicator screening criteria, literature analysis, and research team discussion.

1. Revision of 2 indicators: according to the expert opinion, in identifying swelling due to poor venous return, the combined test of D-dimer and FDP was suggested to exclude the possible interference of heterophilic antibodies to D-dimer detection in some patients due to the use of anticoagulant drugs after TKA. Accordingly, the “D-dimer” indicator was revised to “D-dimer + FDP”. In the preliminary draft, the blood test for coagulation function included only "APTT." However, experts suggested enhancing the test by adding "PT, APTT, Fg, and TT" to comprehensively evaluate the coagulation function. This valuable recommendation was accepted and incorporated.
2. Eight indicators were added: for the secondary indicators of "inflammatory response", experts suggested adding the inflammatory indicators “SAA” and “PCT” to classify the factors causing inflammation, thus distinguishing infectious inflammation from non-infectious inflammation, and adding the “platelet count indicator”. In the tertiary entry for "venous reflux", experts recommend adding a check for “thrombosis” in each vein and a check for “compression of the common iliac vein” to identify lower limb swelling caused by iliac vein compression syndrome (IVCS). Experts recommended adding “popliteal cysts” as a cause of postoperative swelling as a primary indicator and adding “ultrasound” methods for identifying popliteal cysts as a secondary indicator.
3. Thirty-four indicators were deleted: Among the secondary indicators of "inflammatory response", “tumor necrosis factor alpha”, “plasma human alpha defensin 1–3”, and “prostaglandin E2” were deleted due to insufficient specificity or feasibility of detection. Among the secondary indicators of "poor venous return", "blood flow in the common iliac and common femoral veins" was deleted as not meant for diagnosis, while "ultrasonography of saphenous and penetrating veins" was more focused on superficial varicose veins of the lower limbs than on swelling, and thus was also deleted. The "time between the end of ankle pump movement and the return of blood flow velocity to baseline values" was deleted due to the large error in the measurement system. The main indicator "intra-articular capsule synovial fluid" was removed due to insufficient evidence of causing postoperative swelling. Also, its secondary indicators, "ultrasound scan" and "MRI" were deleted due to the high test cost. Among the methods to examine muscle injury and healing, the secondary indicators "MRI", "muscle injury ultrasound scan", "superficial femoral artery", and "blood test SOD1" were deleted because of insufficient specificity. The primary indicator, "ligament injury and healing" was removed because experts felt it was more helpful in assessing postoperative pain and joint mobility but less relevant to swelling.

After the second round of expert consultation, the indicators were revised based on the selection criteria and the study team discussions, after which three indicators were deleted. The tertiary indicator "neutrophil percentage" was deleted because the laboratory experts considered it to be a calculated value of white blood cell count and neutrophil percentage, which is not a measurement parameter. The primary indicator, "popliteal cyst", and the secondary indicator "ultrasonography",which were added in the first round of expert consultation, were deleted because the reasonableness score and coefficient of variation met the criteria for deletion. Also, orthopedic experts suggested that popliteal cysts usually existed preoperatively and did not appear freshly after surgery. The final indicators of the classification and medical tests to identify the cause of lower limb swelling after TKA are detailed in Table 3.

**Table 3:** Indicators of the classification and medical tests to identify the cause of lower limb swelling after TKA

Primary indicators: causes of swelling	Secondary and tertiary indicators: measurement indicators	Reasonableness score (x ± s, points)	Coefficient of variation (CV)
1. Inflammation response		4.82 ± 0.39	0.08
	1.1 Blood tests: routine blood test	4.73 ± 0.45	0.09
	1.1.1 Leukocyte count	4.45 ± 0.78	0.18
	1.1.2 Neutrophil count	4.55 ± 0.66	0.14
	1.1.3 Platelet count	4.82 ± 0.39	0.08
	1.2 Blood test: C-reactive protein (CRP)	4.82 ± 0.39	0.08
	1.3 Blood test: erythrocyte sedimentation rate (ESR)	4.64 ± 0.64	0.14
	1.4 Blood test: Calcitoninogen (PCT)	4.36 ± 0.48	0.11
	1.5 Blood test: amyloid A (SAA)	4.09 ± 0.90	0.22
	1.6 Blood test: Interleukin 1β	4.27 ± 0.75	0.18
	1.7 Blood test: interleukin 6	4.27 ± 0.75	0.18
2. Poor venous return		4.82 ± 0.39	0.08
	2.1 Blood test: D-dimer + FDP	4.82 ± 0.39	0.08
	2.2 Blood test: blood routine (platelet count)	4.82 ± 0.39	0.08
	2.3 Blood test: four coagulation functions (APTT + PT + FG + TT)	4.64 ± 0.48	0.10
	2.4 Color Doppler ultrasound scan: common iliac vein	4.45 ± 0.78	0.18
	2.4.1 Maximum blood flow rate	4.36 ± 0.88	0.20
	2.4.2 Whether under pressure	4.45 ± 0.78	0.18
	2.5 Color Doppler ultrasound scan: common femoral vein	4.64 ± 0.77	0.17
	2.5.1 Blood flow velocity	4.36 ± 1.07	0.24
	2.5.2 Peak velocity of blood flow	4.45 ± 0.78	0.18
	2.5.3 Average velocity of blood flow	4.27 ± 1.05	0.25
	2.5.4 Diameter	4.45 ± 0.89	0.20
	2.5.5 Blood flow	4.18 ± 0.94	0.22
	2.5.6 Thrombus	4.55 ± 0.66	0.14
	2.6 Ultrasound scan: popliteal vein	4.73 ± 0.62	0.13
	2.6.1 Internal diameter	4.36 ± 0.88	0.20
	2.6.2 Thrombus	4.64 ± 0.64	0.14
	2.7 Ultrasound scan: superficial femoral vein	4.64 ± 0.77	0.17
	2.7.1 Internal diameter	4.45 ± 0.89	0.20
	2.7.2 Time, diameter, and regurgitation velocity of the first pair of valves in the superficial femoral vein during calm respiratory state and ValsalVa action	4.45 ± 0.99	0.22
	2.7.3 Thrombus	4.64 ± 0.64	0.14
	2.8 Ultrasound scan: deep femoral vein	4.64 ± 0.77	0.17
	2.8.1 Backflow signal	4.45 ± 0.89	0.20
	2.8.2 Backflow time	4.45 ± 0.89	0.20
3 Joint hematoma		4.55 ± 0.78	0.17
	3.1 Ultrasound scan	4.27 ± 0.75	0.18
4 Muscle injury and healing		4.27 ± 0.75	0.18
	4.1 Ultrasound scan: quadriceps muscle	4.27 ± 0.75	0.18
	4.1.1 Thickness of the rectus femoris, middle femoris, and medial femoris muscles	4.09 ± 0.79	0.19
	4.2 Blood test: serum myoglobin	3.91 ± 0.79	0.20
	4.3 Blood test: serum creatine kinase	3.91 ± 0.79	0.20

## Discussion

The consultation questionnaires in both rounds of this study achieved a 100% valid return rate, indicating a high level of motivation and participation from the experts. These experts come from diverse professional backgrounds and research areas, including osteoarthrosis, rehabilitation medicine, laboratory science, ultrasound, radiology, vascular surgery, orthopedics, and vascular surgery nursing. Their expertise covers a wide range of specialties and encompasses all the tests and examinations included in the indicator system entries. This diverse background provides valuable insights into the various factors contributing to TKA postoperative swelling. With a majority of experts holding doctoral degrees (72.7%), senior titles (81.1%), and having over 20 years of work experience (63.7%), their knowledge and experience in their respective fields are noteworthy. The authority coefficient of the experts was high, with a value of 0.90. The two rounds of consultation, assessed through Kendall's harmony coefficient, revealed a good level of expert coordination, and their opinions tended to be consistent. Following the two rounds of expert consultation, the retained entries met the criteria of importance score > 3.5 and coefficient of variation < 0.25. In summary, this study has constructed the first draft of the indicator system based on a systematic literature search and determined the indicators through the Delphi method. The high authority and motivation of the experts, along with their good coordination and consistency, contribute to the reliability of the indicator system.

### Inflammatory response

In the present study, the inflammatory response was one of the causes of swelling after TKA, characterized by the exudation of extracellular fluid and infiltration of inflammatory cells. While inflammatory response is essential for tissue repair and recovery, excessive intensity or duration can negatively affect surgical outcomes and recovery, leading to complications such as joint stiffness. To address this concern, it is important to accurately identify and effectively manage the inflammatory response [13]. In this study, secondary indicators related to swelling caused by the inflammatory response included white blood cell count, neutrophil count, platelet count, C-reactive protein (CRP), erythrocyte sedimentation rate (ESR), calcitoninogen (PCT), serum amyloid (SAA), interleukin (IL) 1β, and IL-6. These indicators undergo changes at different stages of the inflammatory response process. Inflammatory mediators that are released in response to external stimuli, such as tissue damage and infection, initiate vasodilation, increased permeability, and activation of the monocyte and macrophage systems, causing the leakage of plasma proteins, inflammatory mediators, and cytokines (IL-1β and IL-6) into local tissues, attracting immune cells to the site [14]. Cytokine release influences the synthesis of acute phase reactants (APRs), leading to changes in their blood concentrations. Common APRs include CRP, PCT, SAA, and fibrinogen. Increased fibrinogen levels accelerate ESR. Following the recruitment of immune cells (neutrophils and leukocytes), releasing enzymes, oxygen radicals, and other substances further damages tissue structures and cell membranes, exacerbating the inflammatory response. This process also involves the release of chemokines and inflammatory mediators, attracting more immune cells to the affected tissues. The inflammatory response gradually subsides as the irritants are removed, initiating the repair phase and tissue regeneration. Among the studied indicators, IL-1β and IL-6 serve as early pro-inflammatory cytokines, followed by APR markers such as CRP, PCT, and SAA. Concurrently, ESR increases, along with leukocyte and neutrophil counts. Previous studies have observed that IL-6 peaks in TKA patients one day after surgery and returns to normal within 48 h, exhibiting more rapid changes than CRP and being less affected by patient weight [15]. Studies on the variation in CRP levels following TKA have reported mean CRP values of 57.6 mg/L at 4 days and 5.3 mg/L at 30 days postoperatively. The CRP values at 30 days were not significantly different from those measured before surgery (P = 0.181)[16]. Another study revealed that CRP and fibrinogen peaked during the perioperative period after TKA, reaching 184.3 mg/L and 608.1 mg/dL, respectively. Both indicators returned to baseline levels within 2 and 6 weeks [17]. Considering that the indicators are influenced by the body's immune status and inflammatory response, it is essential to differentiate between the non-infectious inflammatory response caused by TKA surgical injury and the infectious inflammatory response triggered by microorganisms such as bacteria and viruses. This distinction is crucial for guiding subsequent clinical decisions regarding treatment and intervention. Therefore, a comprehensive analysis of these indicators is necessary for a more comprehensive assessment of the inflammatory response status in the organism.

### Poor venous return

In the present study, the poor venous return was considered another cause of swelling after TKA. The endothelial damage of blood vessels after TKA triggered both endogenous and exogenous coagulation processes, resulting in increased platelet count and enhanced synthesis of coagulation factors, thereby inducing a hypercoagulable state. The absence of lower limb muscle compression in the veins further slowed blood flow, leading to venous stagnation and potentially causing lower limb deep vein thrombosis (DVT). Both poor venous return and DVT cause blood pooling in the veins and fluid leaking out of the tissue spaces, eventually resulting in swelling.

The results of the present study that were obtained based on the Delphi method suggest that blood tests and ultrasonography can diagnose poor venous return. Several studies have investigated the predictive capability of D-dimer in detecting DVT after TKA [18, 19]. D-dimer and fibrinogen degradation products (FDP) are plasma markers that evaluate coagulation status and thrombotic risk. Elevated levels of these markers may indicate the presence of thrombosis. Azbo’s [19] research demonstrated a typical pattern of D-dimer changes after TKA, with an initial peak on the first postoperative day, followed by a rapid decline to baseline levels by the third day, and a second peak occurring 15 days after surgery. However, Ye et al*.* [20] observed a second postoperative peak on the 5^th^ day, suggesting its association with the formation of localized microthrombi could aid wound healing. Xinchao et al*.* [18] determined that a D-dimer cutoff value of 0.85 mg/L provided an optimal diagnosis of DVT after TKA, yielding a sensitivity of 78.7%, specificity of 44.1%, and an area under the curve of 0.622 (P = 0.032). Any deviations from normal levels in these indicators may indicate an abnormal blood coagulation condition. In addition, four coagulation tests (APTT, PT, FG, and TT) can be used to assess coagulation factor activity and clotting time, and platelet count to evaluate bleeding and coagulation status, with elevated levels potentially indicating a risk of thrombosis formation. The combined results of these indicators can reflect the patient's coagulation level.

However, it is not sufficient to only consider the patient's coagulation level, as this does not allow for a definite determination of the occurrence of DVT or the presence of venous valve dysfunction causing lower limb swelling. The results of blood tests can only serve as an adjunctive tool for early screening of DVT [21]. Therefore, experts included in this study consistently recommended lower limb venous ultrasound to further determine the underlying causes. Ultrasound, which is noninvasive, nonradiographic, and reproducible tool, can be used to assess the anatomy and hemodynamics of the venous system. Several studies have used color Doppler ultrasound to diagnose DVT after TKA, reporting that the incidence of DVT ranges from 11.77 to 43.75% [21, 22]. It has been suggested that color Doppler ultrasound is a preferred adjunct for DVT detection due to its high incidence and the presence of asymptomatic DVT [23]. Ultrasonography can be used to assess stenosis, obstruction, thrombosis, and the location and size of thrombi in the central venous return pathways, such as the common iliac vein, common femoral vein, and deep femoral vein [23]. For individuals with swelling but no thrombosis, experts recommend evaluating valve function in the popliteal and superficial femoral veins to identify the cause of venous reflux insufficiency. Previous studies have shown that the internal diameter of the popliteal vein is wider in those with DVT after arthroplasty compared to those without DVT, thus suggesting that measuring the internal diameter of the deep veins of the lower extremities may assist in determining DVT occurrence [24]. These findings are consistent with the expert opinion in this study.

Most orthopedic surgeons primarily focus on bones, muscles, and ligaments when considering the causes of post-TKA swelling, with a relatively limited understanding of vascular issues. To gain a more comprehensive and in-depth understanding, this study specifically sought consultation from vascular surgeons who emphasized the potential role of IVCS in causing lower limb swelling. IVCS occurs when the iliac vein is compressed by surrounding tissues, resulting in venous narrowing or occlusion. This obstruction disrupts pelvic venous return, leading to elevated venous pressure and chronic venous insufficiency in the lower extremities, clinically manifesting as lower limb swelling, pain, and varicose veins [25]. Color Doppler ultrasound is the preferred imaging modality for diagnosing IVCS, which enables visualization of the iliac vein blood flow and surrounding anatomical structures [26]. The involvement of vascular surgeons in this study has provided a more comprehensive understanding of the causes of lower limb swelling and valuable insights for diagnosis.

### Joint hematoma

This study suggested that joint hematoma may contribute to lower limb swelling after TKA. Bleeding after TKA primarily results from venous blood loss and osteotomy, with studies reporting significant blood loss of up to 1044–1471 ml within the first 3 postoperative days [27, 28]. In elderly patients (> 75 years old), the blood loss can reach 1741 ml [29], including occult blood loss of approximately 465 ml, which is potentially caused by factors such as hemolysis, blood infiltration into tissues, and postoperative anticoagulant use [27, 28]. Recent research on indwelling drainage also indirectly suggest the presence of joint hematoma [30]. A longitudinal study reported a significant decrease in hemoglobin levels 3 days after surgery (− 4.19 ± 1.18 g/dL) [31]. Additionally, Liu et al. [32] reported prolonged APTT and PT levels and reduced hematocrit on the first day after TKA, revealing important indicators of postoperative hematoma formation. While MRI can examine joint cavity hematoma, its costliness makes ultrasound scans a preferred method to describe the morphology, size, and location of hematoma, as agreed upon by the multidisciplinary experts in this study.

### Muscle injury and healing

The present study suggested that muscle injury and the healing process are potential causes of postoperative swelling. A previous study observed a sharp increase in serum creatine kinase (CK) levels after TKA, peaking on the second postoperative day. Significant correlations were observed between CK levels and postoperative swelling, indicating a relationship between muscle injury and swelling [33]. Another study found significantly larger knee circumference in the group with better quadriceps muscle strength after TKA compared to the group with poorer muscle strength (p < 0.05), proposing a possible association between muscle strength changes and the degree of swelling [34]. Furthermore, moderate quality evidence suggests that knee extensor strength deficits persist in patients after TKA [35]. The quadriceps muscle's maximum voluntary strength (MVS) decreases significantly in the early postoperative period and then gradually recovers linearly, requiring 2–3 years to reach the normal condition [36]. This could mean that prolonged postoperative swelling is associated with the healing process of muscle injury.

Injuries to muscles during TKA mainly include those caused by tourniquet use and those caused by surgical incisions. Tourniquet injury mainly refers to direct mechanical compression injury; however, ischemia–reperfusion injury may be a more important mechanism of injury [37]. The traditional surgical incision for TKA is the medial patellar approach (MPA), which provides good surgical field exposure, but the quadriceps tendon is incised during surgery [38]. A biopsy of the postoperative quadriceps muscle in TKA patients with the MPA approach revealed no change in the morphological structure of medial and lateral femoral muscle fibers, whereas mild to severe atrophy occurred in 88.9% of lateral femoral muscles. This was significantly higher than atrophy in preoperative patients (11.1%) [39], suggesting that surgically induced muscle atrophy exists. Studies on histochemical analysis of muscles after TKA with other methods are still lacking.

Herein, experts agreed that serum myoglobin and serum creatine kinase could be used as markers of muscle damage after surgery, which is consistent with previous studies [40] that have used it extensively to assess the extent of muscle damage after TKA [41, 42]. It has been reported that muscle damage and healing after MRI and ultrasound can identify TKA, but MRI was eliminated from the first round of expert consultation because of its high cost and poor convenience. Experts argued that applying Doppler ultrasound scanning of quadriceps thickness could reflect muscle injury and healing. The quadriceps muscle contains the rectus femoris, lateral femoris, middle femoris, and medial femoris muscles, and previous investigators have reported specific methods and considerations for ultrasound scanning of quadriceps thickness [43, 44].

## Limitations

When discussing the research findings, it is important to acknowledge the limitations of the study. Firstly, the draft of the indicators was primarily derived from the results of the literature search. However, due to time and resource constraints, our literature search did not encompass a broader range of medical databases, which should be taken into consideration in future studies. Secondly, as the sample for this study consisted of Chinese specialists and physicians, there may exist regional and cultural differences in the indicator system. Additionally, while the index system in this study encompassed various causes of postoperative swelling and their related indicators, it does not guarantee that all indicators are equally important and feasible. Therefore, in practical applications, the selection and adjustment of indicators should be made in accordance with the specific circumstances.

## Conclusion

In the present study, we adopted a scientific approach and the Delphi method to identify indicators for the classification and medical tests to identify the cause of lower limb swelling after TKA. The indicators covered the different causes of postoperative swelling and its related tests, thus providing a valuable reference for clinicians to diagnose and treat swelling. Various factors should be considered during application to continuously optimize the indicator system to improve diagnosis accuracy and treatment effectiveness. Yet, future large-scale clinical studies are needed to further verify the practicality and reliability of the indicator system presented in this study.

## Data Availability

The datasets used and/or analyzed during the current study are available from the corresponding author on reasonable request.
